# Palliative care for people living with HIV/AIDS: Factors influencing healthcare workers’ knowledge, attitude and practice in public health facilities, Abuja, Nigeria

**DOI:** 10.1371/journal.pone.0207499

**Published:** 2019-12-31

**Authors:** Whenayon Simeon Ajisegiri, Aisha A. Abubakar, Abdulrazaq A. Gobir, Muhammad Shakir Balogun, Kabiru Sabitu

**Affiliations:** 1 Nigerian Field Epidemiology and Laboratory Training Programme (NFELTP), Abuja, Federal Capital Territory, Nigeria; 2 Department of Community Medicine, Ahmadu Bello University, Zaria, Kaduna State, Nigeria; University of Technology Sydney, AUSTRALIA

## Abstract

**Background:**

Physicians and nurses play vital roles in addressing palliative care (PC) needs of people living with HIV/AIDS (PLWHA). The healthcare workers’ (HCWs) experiences determine the success of palliative care delivery. There is paucity of data on PC for PLWHA. For this reason, we assessed the knowledge, attitude and practice of PC for PLWHA and associated factors among health care professionals.

**Methods:**

We conducted a cross-sectional descriptive study among HCWs in public health facilities in the Federal Capital Territory, Nigeria between February and May, 2017. Multistage sampling technique with proportionate-to-size allocation was used to determine facility sample size and HCWs per professional discipline. **Data** were collected with questionnaires adapted from Palliative Care Quiz for Nursing, Frommelt Attitude toward Care of the Dying and practical questions adapted from PC standard guidelines. Participants' knowledge, attitude and practice were assessed by awarding one (1) point for each correct answer; incorrect or “not sure” answers took a zero (0) score. Correct responses were summed up to get a total score for each participant. Descriptive statistics was done to describe frequencies and proportions displayed on tables. Linear regression was done to determine factors associated with HCW’s knowledge, attitude and practice of PC for PLWHA

**Result:**

With a 100% response rate, the mean age of the 348 participants was 37.5 years (SD: ±8.9), 201 (57.8%) were female, 222 (63.8) were nurses and 230 (66.0%) had a work experience of 10 years or less. Majority of the participants, 310 (89.1%) agreed that palliative care focuses on the relief and prevention of suffering and 319 (91.7%) believe that PLWHA required palliative care. Misconceptions about palliative care include “palliative care is disease-oriented and not person oriented”, 252 (72.6%) believed; “palliative care is concerned with prolongation of life”, 279 (80.6%); and “use of placebos is appropriate in the treatment of some types of pain”, 252 (72.6%). Among the participants, 52% disagreed that “palliative care should be given only for dying PLWHA” while only 18 (5.2%) were right on “family should be involved in the physical care of the dying PLWHA”. Majority of the participants, 292 (84.1%) initiated palliative care discussion during patients’ diagnosis while 290 (83.6%) informed terminally ill patients about their diagnosis. Regarding psychological issues, 22 (6.3%) participants hid the truth from the patients while 196 (56.3%) provided emotional support to the patients. Morphine 240 (69.0%) and Pentazocine 194 (55.7%) were the most commonly used drugs for treatment of severe pain by participants across all centres.

**Conclusion:**

In-service training and undergraduate training on palliative care were associated with knowledge and practice of palliative care for people living with HIV/AIDS. We recommended continuous quality in-service training and education on palliative care for HCWs. While we ensure voluntariness of participation and other ethical principles, the high response rate could be as a result of more motivated health worker than the norm. The results are unlikely to be representative of doctors and nurses in primary health care centres.

## Introduction

Sub-Saharan Africa is the most affected region in terms of Human Immunodeficiency Virus (HIV) infection ad it harbours about 70% of the global population of people living with HIV/AIDS (PLWHA)[1,2]. Nigeria, being the most populous country in Africa with estimated population of 182 million[3] had a national HIV prevalence rate of 3.4% [4] and that of the Federal Capital Territory, Abuja was 7.5% [5] in 2015. The national HIV/AIDS indicator and impact survey (NAIIS) conducted in 2018 estimated both the national and FCT HIV prevalence rate as 1.4% and 1.5% respectively. [6]

The advent of highly active anti-retroviral therapy (HAART) has transformed HIV infection and AIDS to another chronic illness that can be managed but not cured. Despite the use of HAART, PLWHA still continue to die at a higher rate than the uninfected individuals[7]. These among other issues such as HIV-related cancers, co-infections and pains necessitated the need for palliative care as part management of HIV/AIDS [7,8].

“Palliative care is an approach which improves the quality of life of patients and their families facing life-threatening illness, through the prevention, assessment and treatment of pain and other physical, psychosocial and spiritual problems”[9]. It provides pain relief, integrates psychological and spiritual aspects of patients care and offers support system to both the patients and their relatives to live an active live and cope during patient’s illness/death respectively[10]. Very few research studies have been conducted to explore the activities and impact of palliative care for HIV as most attention is placed on ART use[11].

Physicians and nurses play very vital roles in addressing the physical, social and spiritual needs of PLWHA. The health care professionals’ knowledge, attitude, belief and experience go a long way to determine how successful the delivery of palliative care will be [12,13]. In order to strengthen the continuum of care for PLWHA, there is need for considerable investment into research and education on palliative care for health workers who provide services to these population[14].

While several challenges ranging from insufficient funding to weak policies have been attributed to poor development of palliative care implementation and research studies in sub-Saharan Africa [15], countries like India with similar challenges and higher population density have been able to make major breakthrough in the field of palliative care[16]. Majority of the successes in India have been attributed to capacity building of the health care workers and policy makers in the aspect of palliative care. These innovations provide a source of learning to improve palliative care in sub-Saharan Africa.

In Nigeria, palliative care is still evolving despite almost three decades of its introduction. The earlier plan for palliative care intended for cancers and HIV/AIDS was faced with numerous challenges. This made management of cancer cases to face severe limitation but management of HIV cases thrived due to funding from donors[17]. While research studies have been conducted on various aspects of HIV treatment and support, there is paucity of data on palliative care for PLWHA. For this reason, we assessed the knowledge, attitude and practice of health care professionals towards palliative care for PLWHA.

## Methods

### Study design, area & period

We conducted a cross-sectional descriptive study among healthcare workers (doctors and nurses) in public secondary and tertiary health facilities in Abuja, in the Federal Capital Territory (FCT), between February and May, 2017. Abuja is the capital city of Nigeria with a population of 1,406,239. The FCT consists of six area councils and a total of 293 hospitals offering HIV/AIDS services. Of these, government-owned facilities comprised 189 primary, 14 secondary and 3 tertiary health facilities. The health workers all consist of 1,137 doctors, 2,549 nurses and about 900 other allied health staff that offer testing, treatment and prevention services to PLWHA.

We determined the sample size using a prevalence (p) of 30.5% [14], a degree of precision of 5%, a standard normal deviate (Zα) of 1.96. Using the formula for descriptive health studies n =, Z^2^_α_ pq/d^2^[18]; this gave us 326. The estimated minimum sample size was 333 after accounting for 10% non-response rate and correcting for finite population. Total population of doctors and nurses in FCT Health sector is 3686.

### Sampling technique

A multistage sampling techniques was used. Proportionate allocation sampling based on each of the 17 health facilities’ staff strength was used to determine facility sample size. A proportionate-to-size sampling was further used to determine the number of HCWs selected per professional disciplin.

### Study population

Eligible participants were doctors and nurses directly involved in the management of PLWHA or provision of any HIV/AIDS-related services in the public secondary and tertiary health facilities. Allied health staff were excluded as the scope of the study is limited to doctors and nurses only.

Five research assistants fluent at speaking English language and with previous experience in health-related research activities were recruited and trained for a day to ensure standards. All respondents understood and spoke English language and so, there was no need to translate the survey tool into any local language. Data was collected for a period of three months.

### Data collection method, tool and operational definitions

Data were collected using a self-administered questionnaire on “Open Data Kit (ODK Collect)” application on Android mobile phones. The data collection instrument contained four sections. **Section A**: Health workers’ socio-demographic characteristic: age, gender, hospital, qualification, job position, department of work, working experience, training in palliative care. **Section B**: Participants' knowledge was assessed using questions adapted from Palliative Care Quiz for Nursing (PCQN) [14]. One (1) point was awarded for each correct answer while “incorrect” or “not sure” answers took a zero (0) score. Correct responses were summed up to get a total knowledge score for each participant. **Section C**: Attitude was assessed using a 5-item Likert scale with questions adapted from Frommelt Attitude toward Care of the Dying (FATCOD) [14]. It represented participant’s attitudes to a subject scored on 5-point scale– 1 (Strongly Disagree), 2 (Disagree), 3 (Uncertain), 4 (Agree) to 5 (Strongly Agree). Some questions were worded positively and others negatively. The scores of negative items were reversed to calculate the attitude. Overall score was calculated by adding individual’s scores out of 90. A higher score represented a more positive attitude toward palliative care. **Section D**: Practice was assessed through 11 practical questions adapted from previous studies, standard guidelines and literatures related to PC practice[9] [14]. Based on areas of overlap in the different domains, there is lack or conceptual distinctiveness between the attitude surveys and the knowledge and practice measures.

### Data analysis

Data was exported from ODK as Microsoft Excel 2007 version, cleaned and entered into Epi Info version 7 for statistical analyses. Univariate analyses: descriptive statistics was done to describe frequencies and proportions. Linear regression analysis was conducted on demographic variables to determine their association with knowledge, attitude and practice of palliative care for PLWHA.

### Ethical approval

Ethical approval was obtained from the Ethics and Research Committee of the Federal Capital Authority Administration and the selected Health Facilities. Verbal consent was obtained from each respondent before administering questionnaire. Participants’ confidentiality and anonymity was maintained all through the process of the research. The information collected by the study instruments contained no information that can be linked to any participant. All data collected were stored on web open data kit (ODK) server to which only the researchers have access to. The analysis was also combined for all the health facilities and so, not particular individual response can be traced to them or their facilities. They were also informed that they could withdraw from the study at any stage they desire without any consequence.

## Results

The mean age of the 348 participants was 37.5 years (Standard Deviation: ±8.9), and about 43.7% of the participants were within age-group 30–39 years. Majority, 201 (57.8) were female and 274 (78.7) were married, 222 (63.8) were nurses, 231 (66.4%) possess a minimum of bachelor degree and 230 (66.0%) had a work experience of 10 years or less. About 181 (52.5%) of respondents said their undergraduate curriculum consists of palliative care while 70 (20.3%) were not sure. (Table 1).

**Table 1:** Socio-demographic profile of healthcare workers at public secondary and tertiary hospitals Abuja, May 2017.

Socio-demographic variables	Frequency (n = 348)	Percentage (%)
***Age (years)***		
≤29	64	18.4
30–39	152	43.7
40–49	88	25.3
50–59	44	12.6
***Mean age (SD)***: 37.5 years (± 8.9)		
***Sex***		
Male	147	42.2
Female	201	57.8
***Profession***		
Doctor	126	36.2
Nurse	222	63.8
***Marital Status***		
Married	274	78.7
Unmarried	74	21.3
***Highest of academic qualification***		
Diploma (ordinary & higher)	117	33.6
Bachelor Degree	138	45.4
Post-graduate	73	21.0
***Length of post-qualification work experiences (years)***		
≤ 10 years	230	66.9
> 10 years	118	33.1
***Undergraduate curriculum consists of palliative care (n = 308)***		
Yes	181	52.5
Not sure	70	20.3
No	94	27.2
***Received formal in-service training on palliative care (n = 348)***		
Yes	112	36.7
No	246	63.3

The minimum and maximum scores out of 20 on the knowledge scale of palliative care for PLWHA were 1 and 19 respectively with an average score of 11.1 (SD: 3.21). Majority of the participants, 310 (89.1%) agreed that palliative care focuses on the relief and prevention of suffering and 319 (91.7%) believe that PLWHA required palliative care. Most participants, 252 (72.6%) believed “palliative care is disease-oriented and not person oriented”. Others believed that palliative care “is concerned with prolongation of life”, 279 (80.6%); “use of placebos is appropriate in the treatment of some types of pain”, 252 (72.6%); “extent of the disease determines the method of pain treatment”, 283 (81.6%) and that palliative care is “just terminal care”, 165 (47.4%). Majority agreed that “palliative care incorporates the whole spectrum of care: medical, nursing, psychological, social, cultural and spiritual” 285 (81.9%) and that “patient’s family members’ views should be considered in palliative care, 297 (85.6%)” (Table 2).

**Table 2:** Health care workers’ knowledge towards palliative care in public secondary and tertiary hospitals, Abuja, May 2017 (n = 348).

Knowledge area assessed on palliative care	Right option, N (%)
Focus of palliative care	310 (89.1)
Palliative care orientation	70 (20.2)
Palliative care on prolongation of life.	55 (15.9)
Use of multidisciplinary or inter-professional approach	312 (89.7)
Incorporation of whole spectrum of care	285 (81.9)
Identifying of the underlying causes for pain management	284 (81.8)
Primary concern of palliative care	122 (35.1)
Differentiation of palliative Care from hospice Care	134 (38.8)
Differentiation of palliative Care from terminal Care	143 (41.1)
Timing of palliative care institution	140 (40.5)
Method of pain treatment	32 (9.2)
Use of adjuvant therapies palliative care pain management	277 (79.8)
Use of placebos for pain management	252 (72.6)
Involvement of family members in palliative care	297 (85.6)
Palliative care for PLWHA	319 (91.7)
Focus of palliative care	310 (89.1)
Palliative care orientation	70 (20.2)
Palliative care on prolongation of life.	55 (15.9)

Of the 348 participants, the minimum and maximum attitude scores out of 90 were 38 and 81 respectively with an average score of 61.1 (SD: 6.9). About 52% of the participants disagreed (87{25.1%} disagreed and 83 {26.8%} strongly disagreed) that “palliative care should be given only for dying PLWHA” while only 18 (5.2%) were right on “family should be involved in the physical care of the dying PLWHA”. One hundred and fifty-nine (45.7%) participants agreed (making 112 (32.2% the right option) that “they would be uncomfortable talking about death with a dying PLWHA”. (Table 3).

**Table 3:** Attitude of healthcare workers towards palliative in public secondary and tertiary hospitals, Abuja, May 2017 (n = 348).

Statements on attitude of health care workers on palliative care	Right options, n (%)
Palliative care should be given only for dying PLWHA	181 (52.2)
As a patient nears death; the HCW should withdraw from his/her involvement with the patient	277 (80.3)
The length of time required to give care to dying PLWHA would frustrate me	182 (54.3)
Family should maintain as normal an environment as possible for their dying member.	15 (4.3)
The family should be involved in the physical care of the dying PLWHA.	18 (5.2)
It is not difficult to form a close relationship with the family of a dying PLWHA.	62 (17.8)
HCW’s care for the patient's family should continue throughout the period of grief and bereavement	53 (15.2)
HCW’s care should be extended to the family of the dying PLWHA.	46 (13.2)
I am afraid to become friends with PLWHA	238 (68.8)
I would be uncomfortable if I entered the room of a terminally ill HIV/AIDS person and found him/her crying	121 (34.8)
Giving professional care to the chronically sick patient is a worthwhile learning experience	21 (6.0)
Family members who stay close to a dying person often interfere with a professionals' job with the patient.	67 (19.4)
HCW should not be the one to talk about death with the dying person	100 (29.8)
The dying person and his/her family should be the in-charge decision makers	79 (23.0)
I would be uncomfortable talking about impending death with the dying Person	112 (32.2)

Majority of the participants, 292 (84.1%) initiated palliative care discussion during patients’ diagnosis while 290 (83.6%) informed terminally ill patients about their diagnosis. Medical 263 (84.6%) and cultural 94 (30.2%) factors were respectively the most and least considered factors by participants when dealing with terminally ill patients, conditions. Regarding psychological issues, 22 (6.3%) participants hid the truth from the patients, 301 (86.5%) preferred to counsel them while 196 (56.3%) provided emotional support to the patients. Morphine 240 (69.0%) and Pentazocine 194 (55.7%) were the most commonly used drugs for treatment of severe pain by participants across all centres. Majority, 309 (88.8%) perceived the concerns raised by PLWHA or terminally ill patient as their right to treatment while 39 (11.2%), perceived it as threat (Table 4).

**Table 4:** Practice of healthcare workers towards palliative care at public secondary and tertiary hospitals, Abuja, Nigeria—May, 2017.

Practice of palliative care	Options	Agreement N (%)
Initiate palliative care discussion	During diagnosis	292 (84.1)
	When the disease progress	146 (42.1)
	At the end of life	18 (5.2)
Inform terminally-ill patient/PLWHA about their diagnosis	Yes	290 (83.6)
	No	13 (3.7)
	Depending on family’s wish	175 (50.4)
Factors considered when dealing with terminally-ill patient/PLWHA	Spiritual	140 (45.0)
	Medical condition	263 (84.6)
	Cultural	94 (30.2)
	Psychological	148 (47.6)
Ways by which spiritual issues are addressed	Connect with spiritual counsellor	225 (65.0)
	Impose your own view	53(15.3)
	Understand patient reaction	107 (30.9)
Ways by which psychological issues addressed	Emotional support	196 (56.3)
	Counselling the patient	301 (86.5)
	Hiding the truth	22 (6.3)
Cultural issues to be considered during patient care	Truth telling and decision making	194 (56.4)
	Language	128 (37.2)
	Family communication	213 (61.9)
	Perspective on grieving, suffering & death	102 (29.3)
People involved during decision making	Patient	269 (773)
	Family	279 (80.2)
	Other health professionals	196 (56.3)
	My decision alone	12 (3.4)
Perception of concerns or questions raised by PLWHA or terminally ill patient	Patient’s right	309 (88.8)
	Threat	39 (11.2)
	Attention-seeking behaviour	103 (29.6)
	Doubting your professionalism	13 (3.4)
Communication with the family of PLWHA/terminally ill patient depends on:	Family’s ability to assimilate information	266 (76.9)
	Their involvement in decision making	243 (70.2)
	Your willingness to disclose information	46 (13.3)
Commonly use medication for severe pain	Paracetamol	94 (27.0)
	Ibuprofen	112 (32.2)
	Morphine	240 (69.0)
	Codeine	95 (27.3)
	Pentazocine	194 (55.7)

Demographic variables such as Marital status (F = 11.94, p = 0.006); nursing profession (F = 16.37, p = 0.0001) and highest academic qualification (F = 7.38, p = 0.0069) were significantly associated with knowledge of palliative care for people living with HIV/AIDS. However, the co-efficient of determination (R-squared) revealed that these respective variables can only explain 3.3%, 4.5% and 2.1% of the palliative care knowledge. (Table 5).

**Table 5:** Association between demographic variables and knowledge of HIV palliative care among doctors and nurses in public secondary and tertiary hospitals, Abuja, Nigeria—May, 2017.

Characteristics	F-ratio F (1,346)	P-value	t, (95% CI)	R-squared
**Age (years)**				
≤ 40	3.57	0.0596	1.89 (-0.1390–6.9504)	0.0102
**Sex**:				
Female	1.88	0.1712	-1.37 (-5.8027–1.0358)	0.0054
**Marital status**				
Married	**11.94**	**0.0006**	**-3.45 (-11.2171 - -3.0787)**	**0.0333**
**Profession**				
Nurse	**16.37**	**0.0001**	**4.05 (3.6407–10.5271)**	**0.0452**
**Highest academic Qualification**:				
Bachelor degree & above	**7.38**	**0.0069**	**-2.72 (-8.4473 - -1.3529)**	**0.0209**
**Work experience (years)**				
≤ 10	3.36	0.0675	1.83 (-0.2406–6.8795)	0.0096
**Facility type**:				
Secondary	**20.86**	**0.0001**	**-4.57 (-10.9341 - -4.3516)**	**0.0569**
**Palliative care training during undergraduate studies**				
No	**15.77**	**0.0001**	**-3.97 (-10.0089 - -3.3788)**	**0.0436**
**In-service palliative care training**				
No	**5.36**	**0.0212**	**-2.21 (-7.6819–0.6244)**	**0.0153**

Demographic variables such as Marital status (F = 18.16, p = 0.0001); work experience (F = 13.15, p = 0.0003) and health facility type (F = 15.1, p = 0.0001) were significantly associated with attitude of palliative care for people living with HIV/AIDS. However, the co-efficient of determination (R-squared) revealed that these respective variables can only explain 2.1%, 3.7% and 4.2% of the palliative care attitude for PLWHA. On the contrary, sex, profession and in-service training and undergraduate training on palliative care did not have any significant relationship with palliative care attitude towards PLWHA. (Table 6).

**Table 6:** Association between demographic variables and attitude towards HIV palliative care among doctors and nurses in public secondary and tertiary hospitals, Abuja, Nigeria—May, 2017.

Characteristics	F-ratio F (1,346)	P-value	t, (95% CI)	R-squared
**Age (years)**				
≤ 40	**7.57**	**0.0063**	**2.75 (0.6737–4.0548)**	**0.0214**
**Sex**:				
Female	0.41	0.5230	-0.64 (-2.1778–1.1093)	0.0012
**Marital status**				
Married	**18.16**	**0.0001**	**-4.26 (-6.1277–2.2576)**	**0.0499**
**Profession**				
Nurse	0.36	0.5467	0.60 (-1.1711–2.2074)	0.0011
**Highest academic Qualification**:				
Bachelor degree & above	**8.95**	**0.0030**	**-2.99 (-4.2794 - -0.8840)**	**0.0252**
**Work experience (years)**				
≤ 10	**13.15**	**0.0003**	**3.63 (1.4213 4.7896)**	**0.0366**
**Facility type**:				
Secondary	**15.1**	**0.0001**	**-3.90 (-4.7427 - -1.5606)**	**0.0420**
**Palliative care training during undergraduate studies**				
No	0.11	0.7419	0.33 (-1.3532–1.8981)	0.0003
**In-service palliative care training**				
No	0.04	0.8343	-0.21 (-1.8870–1.524033)	0.0001

Demographic variables such as Nursing profession (F = 18.58, p = 0.0001); highest academic qualification (F = 11.73, p = 0.0007); facility type (F = 48.03, p = 0.0001) and in-service training on palliative care (F = 26.19, p = 0.0001) were significantly associated with practice of palliative care for people living with HIV/AIDS. However, age, sex, profession marital status and work experience did not have any significant relationship with palliative care practice for PLWHA. (Table 7).

**Table 7:** Associations between variables and practice of HIV palliative care among doctors and nurses in public secondary and tertiary hospitals in FCT, Abuja, May, 2017.

Characteristics	F-ratio F (1,346)	P-value	t, (95% CI)	R-squared
**Age (years)**				
≤ 40	2.82	0.0942	-1.68 (-5.4658–0.4331)	0.0081
**Sex**:				
Female	0.11	0.7425	-0.33 (-3.3256–2.37293)	0.0003
**Marital status**				
Married	0.55	0.4603	-0.74 (-4.7292–2.1455)	0.0016
**Profession**				
Nurse	**18.58**	**0.0001**	**4.31 (3.3997–9.1061)**	**0.0510**
**Highest academic Qualification**:				
Bachelor degree & above	**11.73**	**0.0007**	**-3.42 (-8.0326 - -2.1719)**	**0.0328**
**Work experience (years)**				
≤ 10	1.75	0.1865	-1.32 (-4.96174–0.9700671)	0.0050
**Facility type**:				
Secondary	**48.03**	**0.0001**	**6.93 (6.6612–11.9404)**	**0.1219**
**Palliative care training during undergraduate studies**				
No	**73.82**	**0.0001**	**-8.59 (-13.7311 –-8.6154)**	**0.1758**
**In-service palliative care training**				
No	**26.19**	**0.0001**	**-5.12 (-10.2645 –-4.5650)**	**0.0704**

## Discussion

The current study found that many of the health workers scored low on the knowledge scale of palliative care and this will adversely impact effective service delivery. This finding is consistent with those from the works of Kassa[14], Gedamu[19], both in Ethiopia and Morsy[20] in Egypt but differs in some other studies conducted in Ethiopia where level of knowledge of participants were high[21,18]. A possible reason for this low score of palliative care knowledge could be because majority of the study participants had not had in-service training on palliative care while just above half had undergraduate palliative care training [20–22].

In-service and undergraduate training on palliative care were significantly associated with the knowledge of palliative care for PLWHA. Lack of any form of undergraduate training or in-service training on palliative care associated negatively with knowledge of palliative care. This is similar to most other studies where training was found to have a positive significant association with good knowledge[14],[19],[22]. Training has been found to increase knowledge of palliative care especially if the content and quality are adequate but could also have no or negative effect on knowledge if the content and quality is poor [19]. The implication of this is that quality, adequate or frequent training on palliative care would promote effective service delivery.

The type of health facilities was significantly associated with knowledge of palliative care Participants in secondary facilities are more likely to score lower on the knowledge scale of palliative care compared to their contemporaries in tertiary health facilities. This may be because secondary facilities do not have sufficient healthcare workers [23] or those with sub-specialties such as providing palliative or hospice care as compared to tertiary facilities [24]. Demand from workload among secondary health facilities staff could also result into reduced attention on palliative care area. It is expected that knowledge of palliative care would be higher in tertiary facility being a teaching hospital and a referral centre. This could be attributed to more academic activities that take place within the tertiary health facilities and they are also generally better equipped than secondary health facilities in terms of service delivery [23]. On the other hand, this finding may simply relates to the knowledge possessed by the individuals rather than the influence of the facilities.

Majority of the participants had some misconceptions about palliative care. The most common ones were that: “PC is disease-oriented and not person oriented”, “PC is concerned with prolongation of life”, “PC is just a terminal care” and that “the use of placebos is appropriate in the treatment of some types of pain”. This could be a demonstration of the poor quality of the training received by participants on palliative care or could be a reflection of how people who need palliative care are being managed. This finding is similar to works done by some other researchers [19],[20],. Poor misconception about palliative care by healthcare workers will negatively influence how care is delivered to the PLWHA in need of palliative care. More so, because of late presentation of most patients to the health facilities and limited healthcare resources, palliative care in Nigeria is limited to end-of-life care rather than the earlier in the disease trajectory[17]. Majority of participants believed that people living with HIV/AIDS actually requires palliative care. This shows there is an understanding of the expansion of the scope of palliative from caring for cancer patients to involving HIV infection and other non-communicable diseases[25].

While demographic factors such as age, marital status, highest academic qualification, facility types and work experience were significantly associated with the attitude of healthcare workers towards palliative care for PLWHA, training and professional professional discipline have no association. Being married was also negatively associated with attitude towards palliative care and different from the findings in the works done in Ethiopia[14]. Highest academic qualification was negatively associated with attitude score and this is similar to the findings from a study conducted in China [26]. The higher the academic qualification obtained, the lower the score on that attitude scale for palliative care. This might be due to the fact that higher qualification might not incorporate the appropriate intervention or care model needed for palliative care delivery [27]. It is important to incorporate palliative care training into academic and career progression as this could improve HCWs’ attitude and lead to better service delivery as well as disease outcome for PLWHA.

About half of the respondents agreed that palliative care should be given to “only dying PLWHA”. This is a misconception as it is expected that palliative care should be given to every person living with HIV/AIDS[9][28–30]. The misconception could be due to the believe that palliative care is the same as end-of-life care. Agreement to this misconception is higher than the findings from other studies where only 15.3%[14], 21.4%[19] and 33.4%[19] are in agreement with this attitude [14],[19].

Over half of the participants said they were uncomfortable with talking about death with a dying PLWHA. This is similar to the study done by Gedamu [19] but higher than other studies done in Egypt [31] and South Africa [32]. This might be associated with our culture, tradition or religious factors that make bring about reluctance in discussing the issue of death with patients. About one-third of health care workers would not want to be assigned to a dying PLWHA. This is lower than the findings from previous studies conducted in Ethiopia, Lebanon and Viet Nam[14],[31,32]. Feeling of incompetence in providing the necessary care for the patients may be responsible from our findings.

Almost a three-quarter of the respondents believed that it is best to change the subject to something cheerful when an HIV/AIDS patient asks “am I dying?” Agreement on this attitude is higher than from other studies.[14],[19]. This again may not be unrelated to religious or cultural issues influencing breaking of bad news to patients. It is advisable to tell dying patients the truth about their condition so that they can make necessary preparation with regards to family, legal and religious issues [33]. as hiding the truth from patients has been considered unethical [34].

Regarding the practice of palliative care for PLWHA, undergraduate training, in-service training on palliative care and highest academic qualification and professional discipline were all significantly but negatively associated. This also similar to studies where training was found to have a significant positive association with practice [14],[19], [22]. This could also be a reflection from increased knowledge associated with quality of the training and adequacy of its content.

Most participants said they would inform terminally ill patients and PLWHA about their diagnosis and this is much higher compare to previous studies in Ethiopia and Lebanon[14],[35],[22]. This might be due to the fact that both doctors and nurses have the responsibility of discussing patient’s diagnosis with them unlike in Ethiopia where it is mainly the responsibility of the doctors[14]. Majority considers medical and about half of the respondents consider spiritual conditions when dealing with terminally ill patients or PLWHA while about two-third connect with spiritual counsellor when handling spiritual matters. This could be due to the Nigerian’s value for religious belief. This is similar to findings from Lebanon and Ethiopia[14],[35].

Morphine and Pentazocine were the most commonly used drugs by respondents for the treatment of severe pain unlike the study in Ethiopia where Paracetamol or ibuprofen were the most commonly used drugs for such[14],[22]. Our finding could be based on healthcare workers’ effort to relief patients’ pain by all means or lack of understanding of WHO analgesic ladders use. However, if appropriately used to relief pain, patients’ quality of life will improve[14]. More so, use of these opioids could also be due to increased access to drugs as there were no restriction to morphine for cancer patients[36] unlike the restriction placed on opioids in Ethiopia[14].

## Conclusion

In-service training and undergraduate training on palliative care were associated with knowledge and practice of palliative care for people living with HIV/AIDS. This could improve the quality of service delivery of palliative care to PLWHA. We recommended that quality in-service training and continuous education on palliative care should be regularly given to improve doctors and nurses’ knowledge and practice.

### Limitations

A limitation of the study is the used of adapted data collection instruments and measures rather than the original. This is a deviation from the originals and could likely interfere with validation as well as the scoring of variables. More so, the palliative care questions on practice were adapted from a previous study and not a validated measure.

Finally, the adapted questionnaire on knowledge was originally designed for nurses. This was used for doctors because we could not find a tool that assessed knowledge of palliative care among doctors as at the time of study. However, review and input by professional colleagues revealed that it could apply to both nurses and doctors in this context with few modifications.

### Strengths

The study adds to the growing body of research which provides evidence that there is inadequate knowledge and practices with regards to palliative care for people living with HIV/AIDS in Sub-Saharan Africa.

The study also brings to bare the need to conduct more research on palliative care for PLWHA as the focus of most palliative research studies conducted in Nigeria have centred mainly on cancer while most HIV-related research studies have been on the aspect of treatment.

Considering that the response rate was 100%, the chances of respondent biases are low. This high response rate could be the effect of relationship established with the institutions during the pre-survey as the research team conducted advocacy visits to the institutions and had several meetings with the heads of institutions and departments. While it is possible that the institutional support and approval may make participants feel compelled to participate in the study, we ensured that voluntary participation and other ethical principles were adhered to. Research assistants also followed participants up through repeated personal contact.

The responses of the participants may possibly be an over-estimated knowledge and practice given that the healthcare workers are those involved in the management of PLWHA and are likely to have been more motivated health workers than the norm. The responses may not likely be representative of healthcare workers in primary health care settings and among healthcare workers who are not involved in management of PLWHA within the participating health facilities.

## Supporting information

S1 File The minimal data set for analysis. (XLS)
